# The best laid plans: How do adopted city sustainability goals influence site-level action in urban forestry?

**DOI:** 10.1007/s13280-025-02247-0

**Published:** 2025-09-26

**Authors:** Corinne G. Bassett, Susan D. Day, Cecil C. Konijnendijk, Lara A. Roman, Chanel K. Yee, Kai M. A. Chan

**Affiliations:** 1 Nature-Based Solutions Institute (Dutch Office), Eerste Muntmeesterslaan 17, 3541GB Utrecht, The Netherlands; 2 Faculty of Forestry, University of British Columbia, 2424 Main Mall, Vancouver, BC V6T 1Z4 Canada; 3 USDA Forest Service, Pacific Southwest Research Station, 4955 Canyon Crest Dr., Riverside, CA 92507 USA; 4 Institute of Resources, Environment, and Sustainability, University of British Columbia, 429-2202 Main Mall, Vancouver, BC V6T 1Z4 Canada

**Keywords:** Climate action plan, Multi-objective management, Management paradigm, Urban sustainability, Urban tree planting

## Abstract

Cities increasingly hope to mobilize nature-based solutions in urban sustainability planning because of their wide-ranging ecosystem services. In this critical moment, where failure due to poor implementation could lead policymakers to turn away from nature-based solutions, we investigate the case of urban forestry and ask: how do strategic-level sustainability goals influences site-level decision-making? We conducted semi-structured interviews with 20 urban foresters leading advanced programs at municipalities with adopted sustainability goals, framed as ecosystem services, across the US and Canada. Day-to-day, site-level decision-making focused on advancing a “more, bigger trees” paradigm with the justification that this would deliver increased ecosystem services in a general sense and thus contribute to city goals. Our analysis and results suggest that urban foresters are more guided by a shared sense of the greater purpose of their work to serve urban communities than city plans, which yields actions usually aligning but sometimes conflicting with specific strategic-level goals.

****Graphical abstract**:**

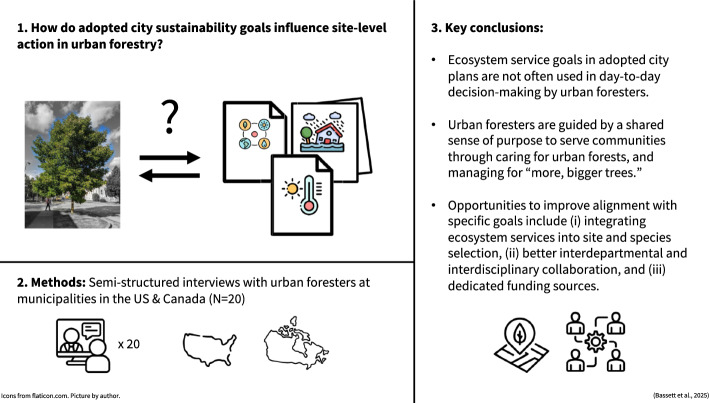

**Supplementary Information:**

The online version contains supplementary material available at 10.1007/s13280-025-02247-0.

## Introduction

City leaders around the world are seeking to mitigate rising urban temperatures, improve stormwater management, and generally meet critical sustainability goals in the face of mounting environmental threats, by adopting ambitious plans that often rely on nature-based solutions such as urban forests (Stone et al. 2012; Keith et al. 2023). Successful implementation of nature-based solutions requires engaging specialized expertise from multiple disciplines and a strong commitment to understanding and making decisions based on local social and ecological conditions (Frantzeskaki 2019). However, it is uncertain whether these city plans will actually lead to their urgently needed outcomes (Stone et al. 2012), and in particular, if site-level decision-making around nature-based solutions is sufficiently aligned with these plans. Many of these plans rely, in part, on urban forests—the communities of trees and associated vegetation in urban areas—as a nature-based solution because of their dominance as a system in many cities and the diverse ecosystem services they can provide. Initial evidence suggests significant gaps in alignment between urban forest management decisions at the site-level and sustainability goals set at city or regional levels (Ordóñez Barona et al. 2024a), such as those set forth in climate action plans (Cheng et al. 2021).

One concept put forward to aid in bridging such divides in planning and decision-making is the concept of ecosystem services, which has been widely integrated into urban planning, urban forestry, and in study of nature-based solutions generally, in large part because of its ability to connect disciplines around shared language about the contributions of ecosystems to human needs (Gómez-Baggethun and Barton 2013; Lam and Conway 2018; Escobedo et al. 2019). Nonetheless, this advantage is not without pitfalls, as scholars have long raised concerns that orienting ecosystem management toward specific ecosystem services encourages a reductionist and human-centered approach, blinding decision-makers to the full complexity of ecosystems (Norgaard 2010; Luck et al. 2012; Klain et al. 2014). While natural resource managers such as urban foresters are typically highly knowledgeable and care deeply about the ecosystem services of the resource they manage, it is unknown how or if these professionals connect their management decisions to the adopted sustainability goals of their city (Ordóñez et al. 2019), and if municipal sustainability planning is successfully integrating such professionals and their specialized knowledge in service of critical goals.

### Operationalizing the concept of ecosystem services in practice

Understanding the concept of ecosystem services, broadly defined as the benefits to people from natural ecosystems (Daily 1997, Millennium Ecosystem Assessment 2005), is key to understanding the evolution and practice of nature-based solutions such as urban forestry (Dorst et al. 2019; Escobedo et al. 2019). The ecosystem services concept has strongly influenced professional discourse and identity in fields such as urban planning and urban forestry, including in the US and Canada, as evidenced by its increased inclusion in municipal plans spanning land use to urban heat to urban forest management (Thompson et al. 2019; Keith et al. 2023; Ordóñez Barona et al. 2024b). An original promise of the concept was not just to communicate the values of natural ecosystems, but to be used to develop ecosystem management objectives and metrics (Daily et al. 2009; Hansen and Pauleit 2014; Arkema et al. 2015). In particular, as ecosystem management actions have long been shown to present tradeoffs and synergies in the provision of multiple ecosystem services (Chan et al. 2006; Naidoo and Ricketts 2006; Nelson et al. 2009), calls have been made not only to justify the conservation and restoration of ecosystems but to use ecosystem services framings in urban planning to better manage these tradeoffs (Cortinovis and Geneletti 2019; Thompson et al. 2024). This drive to operationalize ecosystem services in the planning process relates to its potential to serve as a boundary object, a concept which can connect siloed disciplines around shared language, and facilitate decisions toward goals which benefit society as a whole (Abson et al. 2014; Carmen et al. 2018).

One way that ecosystem services can connect different systems is by parsing out specific ecosystem processes, and thus connecting those who manage ecosystems from siloed disciplines, such as urban forestry, with other fields, such as public health, stormwater management, or climate science (Jackson et al. 2013; Abson et al. 2014; Thompson et al. 2024). The ecosystem service cascade model (Polasky 2011, Potschin and Haines-Young 2011), a widely adopted framework for conceptualizing ecosystem services, describes these connections as flows from ecosystems to people captured by a cascade of processes from ecosystem structure (e.g., canopy cover) to function (e.g., transpiration), to service (e.g., cooling effect), to benefit (e.g., reduced incidence of heat strokes), and to value (e.g., health care savings). Ecosystem structures have many functions, services, and benefits, each with their own proxy indicators. Framing objectives as either “reduce urban heat” (i.e. service focused) or “preserve large trees,” (i.e. structure focused) can impact the actions and the type of expertise required to align with such goals (Olander et al. 2018; Bassett et al. 2024). Though the concept of ecosystem services could be used as a boundary object or connector, especially between urban forest management and adopted city goals which urban forests can contribute toward, it is unclear if the concept is being used as such.

Challenges in using ecosystem services for both planning and goal-setting include, among many issues, agreeing upon methods to define and assess ecosystem services across stakeholders (Carmen et al. 2018) and the tendency to undervalue cultural benefits, which are difficult to quantify (Chan et al. 2012; Daniel et al. 2012). In addition to these technical challenges, concerns have been raised that thoroughly embracing ecosystem services in ecosystem management would lead to only recognizing the instrumental values of nature, neglecting its intrinsic and relational values, and encourage a reductionist, anthropocentric, and approach to management (Norgaard 2010; Chan et al. 2018; Matzek and Wilson 2021). Understanding the ability for fields like urban forestry to be mobilized as nature-based solutions for strategic sustainability goals, requires understanding both the biophysical processes and conditions for goals to be met and how the professionals who will implement such interventions conceive of their own objectives and impacts of their actions.

### Ecosystem services and the profession of urban forestry in the US and Canada

Simultaneous to the rise and many evolutions of the concept of ecosystem services in the past decades, and critical to understanding the perspectives of practitioners, has been the development of urban forestry as a standalone profession (O’Herrin et al. 2023). Urban foresters include professionals who work in many sectors, including governments (primarily municipal), private industry and consulting, non-governmental organizations, and academic and educational institutions (Day et al. 2022). The professional identity of urban foresters, while still being defined, is uniting around a shared mission to manage trees and forests in urban areas as an essential service to society, because of their many ecosystem services (O’Herrin et al. 2020).

Broader research has found that professions can promote the development of “group-based values” among their members, influencing their decision-making and priority setting (Breed 2022). As the professional formation of urban foresters formalizes, there is significant evidence that ecosystem services is a core concept that professionals are expected to be versed in, such as its inclusion in key textbooks (Miller et al. 2015; Ferrini et al. 2017) and credential knowledge areas (Lilly et al. 2022; Society of American Foresters 2024). A survey of urban forestry educators in the US and Canada even found ecosystem services ranked as a top five knowledge domain (out of 26 total) for their programs (Barron et al. 2025). Training created by and for urban forestry professionals emphasizes that their central role is to maximize the ecosystem services of trees in order to benefit society (O’Herrin et al. 2020; Roman et al. 2021). How this shared mission influences the management decisions of urban foresters is critical to understanding the alignment of their decisions with city goals.

Though urban foresters are very familiar with the concept of ecosystem services and even see it as a dominant paradigm in their field (Silvera Seamans 2013; Young 2013; Barron et al. 2025), initial inquiries of similar urban foresters in Australia and the UK suggest that they do not usually employ a formal ecosystem service-based management approach, that is, the concept has not been operationalized beyond use in advocacy to direct decision-making (Davies et al. 2017; Roy et al. 2017). Instead, urban forest management goals tend to relate to ecosystem structure, such as increasing tree abundance (Ordóñez Barona et al. 2024b) or species diversity (to address socioecological resilience, see Huff et al. 2020), rather than specific ecosystem services (Roy et al. 2017). One exception is the emphasis on managing disservices, especially the risk of damage to people and property from falling trees and branches through regular inspection and pruning, termed tree risk management (Hauer and Peterson 2016; Koeser et al. 2016). The relationships between decision-making processes and program objectives in urban forestry are poorly understood (Ordóñez et al. 2019). The popularity of i-Tree, an ecosystem services modeling suite of tools developed by the United States Department of Agriculture (USDA) Forest Service (Nowak 2020), and the rise in city plans with ecosystem service goals (Thompson et al. 2019; Angelo et al. 2020), suggest that urban forest management objectives are changing to focus more on ecosystem services. This change could alter benefits delivered to urban communities, as there is strong evidence that decisions throughout the urban tree management life cycle present tradeoffs in the provision of multiple ecosystem services and minimization of disservices (Roman et al. 2021)—spanning design (Bruner et al. 2023), planting (Bodnaruk et al. 2017), maintenance (McPherson et al. 2015), and eventual end of life and removal (Dickie et al. 2014). However, despite the theoretical ability to make site-level decisions so they explicitly align better with city goals, especially when framed as ecosystem services that urban forests can provide, there are many barriers to doing so, including urban forestry’s current isolation from other disciplines (Vogt 2018).

### Study Background and Research Questions

Achieving city sustainability goals with nature-based solutions, such as urban forests, requires the successful alignment of decision-making at multiple levels and bridging barriers to interdisciplinary collaboration. We addressed these fundamental challenges to urban sustainability through an investigation of urban forest management, and the role of the concept of ecosystem services in facilitating alignment of strategic goals and site-level decisions. To do so, we investigated cities working toward sustainability goals, as evidenced by their inclusion in any adopted municipal plan, focusing only on those framed as ecosystem services that urban forests provide (which we hereafter refer to as ecosystem service goals). The scope was narrowed to street trees for comparability across cities because the trees which municipalities manage vary significantly in type from natural forest patches to parks to streets. This research aims to fill existing gaps in understanding how ecosystem service goals guide decision-making of urban foresters, and more generally, if the concept can be used to connect actions at small scales in service of large-scale goals.

In light of these fundamental questions, we investigated the following research questions and objectives in the context of urban foresters:

1. To what extent is site-level decision-making influenced by adopted municipal ecosystem service goals?
2. What are the current challenges and best strategies in aligning site-level decisions to contribute to municipal ecosystem service goals?
3. How is the concept of ecosystem services used to bridge site-level decisions and strategic goals?

## Materials and methods

### Approach

We conducted semi-structured expert interviews to investigate specialized knowledge within the field of urban forestry, as well as to uncover both explicit and implicit understandings, opinions, and preferences (Döringer 2021). Study design and analysis followed an “iterative phronetic approach” (Tracy 2019), an approach developed and inspired by methods such as thematic analysis and grounded theory (more detail in 2.4). This is a reflexive and inductive approach which allows researchers to not only construct theory but also to inform and guide practice and action, making it well suited for the aims of this study. This study was part of street tree futures, a multi-year research-practice partnership led and advised by a committee of researchers and practitioners.

### City and participant selection

We interviewed urban foresters employed at an array of cities in the United States (US) and Canada, varying in population and geographic location, with particularly advanced urban forestry programs and adopted ecosystem service goals (see below criteria). An initial list was created utilizing two recently published lists of cities with urban forest management plans in the US (Grant et al. 2022) and Canada (Cheng et al. 2021). Additional municipalities known to have advanced urban forestry programs were added by the co-authors and the Street Tree Futures project practitioner advisory committee, composed of members of urban forestry NGOs and private industry organizations with national reach in the US and Canada. The initial list was then evaluated against inclusion criteria (Table 1) to create a list of eligible cities, using information from city websites and online searches.

**Table 1:** Participant inclusion criteria. The sampling approach first targeted municipalities meeting the below criteria, and then participants employed at those cities who met additional criteria

**Municipality inclusion criteria**
• Located in the US or Canada • An advanced urban forestry program indicated by having at least two out of three of the following: (i) an urban forest management plan or similar strategy document published within ten years of the study start (Jan 2013- Jan 2023), (ii) Tree City USA (Arbor Day Foundation 2024a) or Tree City of the World recognition (Arbor Day Foundation 2024b), (iii) a full or partial street tree inventory • At least one formally identified ecosystem service goal within an adopted municipal plan such as those related to climate change mitigation, urban heat, stormwater management, biodiversity, or human health, with the potential for application of urban forests as a strategy to address such goals (see Supplement; Table S1 for examples) • The authority to manage street trees under its jurisdiction
**Participant inclusion criteria**
• Responsible for making decisions, or significantly informing the decisions, about municipal street trees in at least one (but preferably most) of the following phases: planning/design, planting, maintenance, and/or removal • Example titles include terms such as: manager, director, chief, lead, combined with either urban forestry or arboriculture • At least five years’ work experience in relevant fields • Employed at their current city for at least 6 months (i.e., familiar enough with their municipality to speak on how decisions are made there)

A final list of 54 eligible cities was categorized and sorted by country, geographic area, and population. A random number generator was used to assign an outreach order, spread across the categories, with the goal of achieving evenness in representation and removing bias from the selection process. Potential eligible participants in each of the 54 eligible cities were identified by city website searches, recommendations of the project’s research and practitioner advisory committees, and through requests for recommendations via contacts at urban forestry departments found on city websites. The goal of recruitment was to identify employees with both managerial and operational duties, i.e., those involved in operational decision-making about street trees and in enough of a leadership position to participate in their city’s planning processes (Table 1).

Prospective participants from the first 20 cities from the order created by the random number generator were emailed. They were asked to sign a consent form and schedule a one-hour interview within two weeks, after which a follow-up email was sent. When initial contacts did not respond to follow-up emails, additional prospective participants from remaining eligible cities were contacted, aiming for evenness with respect to population and geographic region. Twenty interviews were chosen as the initial goal, based on numbers of subjects in similar studies conducting interviews with urban forest professionals (Davies et al. 2017; Sax et al. 2020; Powning et al. 2024; Quinton et al. 2024). After the 20 interviews, it was assessed that saturation had been reached (Guest et al. 2006; Tracy 2019) and there would likely be limited new information gleaned from additional interviews.

**Fig. 1:**
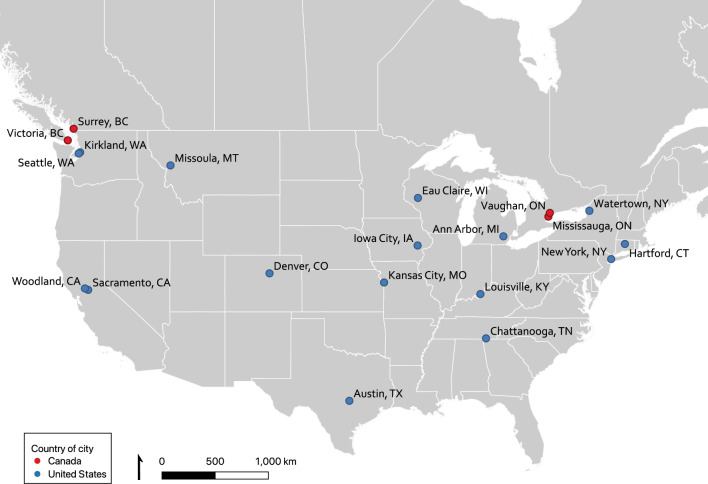
Map of cities with participating municipal employees. Points labelled with city name and state/province abbreviations

Twenty participants were interviewed, each employed by a different city. Cities ranged in population and geographic area (Table 2; Fig. 1).

Overall, participants were a highly knowledgeable and experienced group of professionals, ranging from mid-to-late career professionals (over half had more than 15 years of work experience in urban forestry; the youngest participant had seven years of experience). All held degrees in relevant fields (incl. forestry, natural resource management, etc.) and/or professional credentials (e.g. International Society of Arborists Certified Arborist). Most participants employed in parks and recreation, or public works departments were in positions where they directed staff performing street tree maintenance (performed by crews on staff or by contractors), though their interaction with this level of activity varied based on city size. Participants employed in planning departments were more often involved in street tree management via code enforcement of tree ordinances/bylaws or development plan approval, though these participants strongly identified as urban foresters and were engaged with the tree maintenance procedures in their cities. All participants were employed at municipalities whose official names contained the term “city,” regardless of size. For brevity, we will thus use the term “city” throughout this paper.

**Table 2:** Employment and education background of semi-structured interview participants (n = 20) by city population size. City population classifications: small < 100 000; 100 000 < medium > 700 000; large > 700 000

	City size					
	Small (n = 7)		Medium (n = 8)		Large (n = 5)	
	*Count*	*%*	*Count*	*%*	*Count*	*%*
**City department**						
Parks and recreation	5	71.4	4	50.0	3	60.0
Public works	0	0.0	3	37.5	1	20.0
Planning	2	28.6	0	0.0	0	0.0
Forestry	0	0.0	1	12.5	0	0.0
Transportation	0	0.0	0	0.0	1	20.0
**Education level**						
Masters	2	28.6	2	25.0	2	40.0
Bachelors	5	71.4	5	62.5	2	40.0
Likely bachelors	0	0.0	1	12.5	1	20.0
**Time in urban forestry (years)**						
5–10 years	2	28.6	1	12.5	1	20.0
11–20 years	1	14.3	3	37.5	1	20.0
> = 21 years	3	42.9	1	12.5	3	60.0
Not mentioned	1	14.3	3	37.5	0	0.0
**Time employed at city (years)**						
< = 5	3	42.9	4	50.0	1	20.0
6–20	3	42.9	2	25.0	2	40.0
> = 21	1	14.3	2	25.0	2	40.0

### Interviews

Semi-structured interviews were organized into four sections. In order, these were opening (ex. “What are your primary responsibilities and tasks in your current role?”), generative (ex. “Do you think it is part of your position’s job duties to manage trees for any *specific* ecosystem services?), directive (ex. “In your decision-making, how important is this [ecosystem service] goal compared to your previously stated street tree management objectives?”), and closing sections (ex. “In an ideal world, what do you think are the best practices for cities to align street tree management actions to contribute to city-level ecosystem service goals?”) (Tracy 2019) (see Supplement; Table S2 for complete interview guide).

All interviews were conducted by the lead author, a doctoral student with prior professional experience working in urban forestry and arboriculture. The interviewer positioned themselves as a “co-expert” (Döringer 2021), and began interviews by briefly introducing their background in the field and journey to the chosen research questions, establishing a rapport that could be characterized as both peer-to-peer and expert-to-student.

Twenty interviews of 34 to 100 min (with most lasting around 60 min) were conducted and recorded via video-conferencing software (Zoom 2024) in February and March of 2023. Interviews were transcribed using Otter.ai Pro 2023 (Liang and Fu 2023) after which produced transcripts were manually edited for comprehension (e.g., mis-transcribed words, speaker assignment).

### Analysis

Analysis followed an iterative phronetic approach, chosen for its suitability for research closely tied to a pre-identified research question or problem and intending to “shed light on an issue or open a path for possible social transformation” (Tracy 2019). The lead author conducted primary (or “open coding”) and secondary cycles of code development, which led to an initial codebook of themes. Through memo-writing by the lead author, the process of refining codes was guided by iterative, continuous reflection on the themes which recurred in the data and the dialectical relationships and tensions between those themes and the original research questions (Srivastava and Hopwood 2009; Tracy 2019).

Comparison coding then was conducted with a second coder (also a co-author), where both coders analyzed two randomly selected transcripts using the initial codebook, and together further refined it through in-depth discussion of agreements and discrepancies. This final shared codebook was then utilized by both coders to independently code remaining transcripts (n = 18), with one coder coding odd numbered transcripts, and the other coding even ones. The coders frequently conferred with each other to refine or add to the codebook, as necessary. All analysis was conducted in NVivo 14 (NVivo 2023). As a result of the analysis, we offer the frequencies of participant interviews in which themes were found to give insight into their relative prominence among our interviewees. However, this qualitative methodology is not designed to assess the frequency of specific opinions within a sample population, so it is important not to over-interpret or project conclusions based on the numeric frequency of certain codes. Coding matrix queries were conducted in NVivo to explore the frequencies of codes across participant and city factors (Table 2); however, no notable trends were found and are thus not reported in the Results section.

## Results

### Context of urban forest management objectives

Participants were at the intersection between high-level strategic planning and detailed operational responsibilities, such as managing service requests from residents and equipment needs. When asked about the most important objective that drives street tree management decision-making, participants described urban forest health and growth (n = 11), tree risk and public safety (n = 9), and serving the public (n = 8) as their most important objectives. Many participants were unable to choose a single objective as most important in all situations and described the purpose of their urban forestry programs as blending tree health, risk management, and benefit provision.

*“Keeping trees alive and providing benefits, safely, for as long as possible.” (P13)*

*“The objective is to keep the public safe, that’s first and foremost. But then also to keep our urban forests healthy.” (P2)*

When asked if it was part of their job duties to manage trees for any *specific* ecosystem services, just over half said no, not directly, but that they manage for ecosystem services broadly (n = 11), while the other half of respondents said yes, often highlighting several services they were focused on (n = 9).

*“It is my opportunity, and in many cases, my pleasure to manage trees for their ecosystem benefits. It is not necessarily my responsibility, or within my power, to manage the full range of environmental benefits that trees and ecosystems can provide.” (P19)*

*“Yes, I would say for sure stormwater mitigation. Working with our interdepartmental partnerships in stormwater mitigation is a huge goal.” (P11)*

### Strategies and challenges to align street tree management with specific city ecosystem service goals

At times, it was difficult to distill participants’ strategies to align street tree management with specific ecosystem service goals, because they often viewed any actions toward the purpose of their programs to improve tree health and longevity as being in alignment with these goals. Specific strategies and challenges are described in sub-section "Strategies and challenges to align street tree management with specific city ecosystem service goals", and this tension led to the development of the themes explored in subsequent sub-section "Perceptions of ecosystem services, street tree management, and city goals".

#### Strategies to align street tree management with specific city ecosystem service goals

All participants described strategies to align site design and species selection (n = 18) with specific city ecosystem service goals and using data or tools to support those decisions (n = 18). For example, the Tree Equity Score, a spatial index of socio-environmental factors (American Forests 2024), was frequently referenced as a potential strategy to align new tree plantings with a city’s goals to reduce urban heat and better serve low-income communities.

*“First, it’s going back to GIS and like ‘hey, is this an area of high need of trees, based on all the factors, low canopy, high urban heat, underserved neighborhood?’” (P1)*

Factors within their municipality were also seen as fundamental, including support from elected officials (n = 14) and the alignment of existing urban forest plans and policies (n = 14). Regular, formal, or informal communication with other city staff, including building inter departmental relationships, was also described as a key to advancing their city’s ecosystem service goals (n = 13).

*“There’s a lot of change going on in local government, now that the city council just adopted a new climate lens, which will factor into all decisions made within city purchasing, prioritization of projects, all these things.” (P3)*

*“I just met with our transportation engineers yesterday about, in our big intersections, finding ways to slow traffic, pull out impervious surfaces, put in trees and other vegetation to help with the heat island effect and to collect stormwater, and all these other things. So, yeah, it’s in every single department here.” (P13)*

About half of participants described partnership with an organization specializing in a specific ecosystem service goal (n = 11), either internally, such as their city’s water department, or externally, such as an NGO focused on food security, as a strategy. Enabling community stewardship and support (n = 12) were also seen as key to reaching goals.

*“There’s a group called [city name] Urban Gardens [...] and we’re actually starting to plant apple trees and other things.” (P10)*

*“We partner and support each other with the department of utilities who handle stormwater. […] on one hand, I’m working with a tree, but I’m promoting a goal that is actually somebody else’s goal.” (P19)*

Another strategy was to follow models from other cities (n = 7), for example, through utilizing design specifications for trees in new projects geared toward a specific ecosystem service. Strategies like allocating funding (n = 10) or creating a new staff position (n = 5) described ways to direct work toward one specific goal or another. Nonetheless, for almost all participants, their program’s current goals to improve urban forest health and protect public safety remained the top priority.

#### Challenges to align street tree management with specific city ecosystem service goals

The challenges to align street tree management with specific city ecosystem service goals were in many ways the “other side of the coin” of the strategies previously described. While departmental collaboration had been previously described as a strategy, departmental silos were a frequently described challenge to advancing ecosystem service goals in urban forest management (n = 10). Institutional arrangements, and the position of urban forestry within them (e.g., in a parks department, public works, or planning department) also affected priorities.

*“From a park management standpoint, I mean, we do manage for culture, and you know, pollinator friendly, all those kinds of things. But that’s, um, like a park maintenance priority, that’s not really an urban forestry priority.” (P3)*

Insufficient resources, such as lack of funding (n = 14) and problems with staffing (n = 11) were also commonly cited challenges.

*“We’re not in a position to have a lot of discretion in what or how we’re making decisions, because we have a very finite amount of resources. So, you know, we have to do what makes sense in every situation. Sometimes that doesn’t match with our overarching ecosystem goals.” (P3)*

*“I know what I want out there, I know what the Climate Action Plan is asking for. Money is usually the biggest problem.” (P20)*

Many also described their current operational procedures for street tree management as not allowing for strategic decision-making (n = 9). For example, being solely service request based (which was a common but not universal setup) also affected their ability to take on higher level objectives.

*“So, in urban forestry, the way I’m set up as a maintenance organization, is not well geared to track overall climate change or the benefits of the trees I’m planting or maintaining. So, I would be poorly suited to meet the requirements and restrictions of [climate mitigation specific funding].” (P19)*

Current knowledge and preferences of both city staff and the public were also seen as challenges by some interviewees. While some described their cities’ desire for new, perhaps “flashier,” strategies to reach goals such as pop-up parks or new capital improvement projects, instead of investing in maintenance of existing trees as an impediment (n = 5), inertia was also brought up as preventing changes to their programs (n = 3). A lack of evidence-based guidelines for aligning management with particular ecosystem services was also described (n = 4). For example, one participant described not knowing of any local guides for which species were best suited to help achieve specific objectives.

However, the most common challenges related to the built environment limiting street tree management choices (n = 17). For example, when harsh growing conditions (e.g., insufficient growing space, compacted soil) limit the survival potential of many native trees, it can be infeasible to plant them in service of a biodiversity goal.

Competing land use priorities (n = 9), such as whether to develop land for new housing vs. preserve native habitat, were reported as limiting street tree management choices and efforts to achieve ecosystem service goals by reducing available space for existing and new trees. A particular challenging example of competing priorities was in considering the role of urban forestry in reaching biodiversity goals for cities in non-forested biomes:

*“Parks and Rec is really big on natural grasses [...] We’re a novel ecosystem within the ecoregion of shortgrass prairie [...] so, the push isn’t to plant more trees, the push is actually to return it back to its original state. I go home with a lot of headaches sometimes because on one hand politicians want trees, trees, trees, but then it’s like, like the old saying, don’t listen to what they say, but watch what they do. So, they say trees, trees, trees, but they’re pushing for more natural landscapes.” (P10)*

### Perceptions of ecosystem services, street tree management, and city goals

Complexities related to the concept of ecosystem services strongly influenced participants’ perception of whether their work aligned with city ecosystem service goals, and even more fundamentally, how they viewed the objectives of the urban forestry programs they managed. The following four themes were constructed to describe the current paradigm of street tree management and alignment with municipal sustainability plans as reflected by these interviews.

#### Understanding of urban forest ecosystem service provision

All 20 participants had high familiarity with the concept of ecosystem services and were well versed in the many possible benefits of urban trees. Participants repeatedly emphasized that their job was to increase the longevity of trees (n = 15), grow trees of larger stature (n = 12), and grow more trees (n = 10), and that these outcomes would all result in “more” ecosystem services.

*“Maximize the size of tree that you can get in any appropriate location. Because when you’re doing that, you’re inherently setting yourself up at least for maximum ecosystem benefits when you’re following right tree, right space, all those things and also planting as many trees as you possibly can in those spaces” (P3)*

This was often expressed through the idea that managing for overall tree health and increased canopy coverage would result in more ecosystem services (n = 14), and thus, actions taken to increase these structural goals were aligned with ecosystem service goals.

*“When I said I focus on canopy coverage, that’s going to hit all the climate goals about heat island effect, about stormwater retention, about habitat.” (P19)*

At the same time, participants also articulated how street tree management choices could result in different ecosystem services (n = 8), especially through species or site selection. They also described how certain urban forest types were better at providing, or designed to provide, different ecosystem services (n = 12), for example, describing that the main roles of street trees were to provide shade and beauty for pedestrians while natural forested areas in city parks were better for providing wildlife habitat.

#### Role of the ecosystem services concept in street tree management

Participants primarily described using ecosystem services as a justification for funding and the existence of their street tree management programs (n = 15) by, for example, advocating for their budget to city leadership or using ecosystem services to make the case for tree preservation to the public. Over half described having an ecosystem service assessment and valuation, either using i-Tree (Nowak 2020), or through a tree inventory software tool with embedded ecosystem service estimates. By far the most dominant ecosystem service assessment tool referenced was i-Tree. Other software mentioned by participants utilized i-Tree models for ecosystem services estimation, such as TreeKeeper, a proprietary tree inventory and work management software (Davey Tree 2024) and the Tree Equity Score, a web-based tool which, though focused on characterizing distributional equity of canopy cover, uses i-Tree to estimate the benefits of increasing tree cover (American Forests 2024). Most believed that describing—and even better, quantifying—the benefits of trees was the best way to gain support for urban forest management.

*“When you talk about tree quantities, and even canopy cover percentages, as, you know, the sort of measurables, it just gets lost for people. Like, people don’t give a s--- whether you have a 25 or 35% canopy cover percentage, right. But if you tell them that we were able to, you know, offset a million-and-a-half dollars worth of carbon, or, you know, here is the impact that we’ve had on our stormwater management system, or you know, some of these other sorts of ways to tell our story, that’s where the real benefit of that stuff is.” (P16)*

An advocacy strategy of describing one specific ecosystem service to get the attention of a specific audience also frequently came up in the interviews (n = 12). Nonetheless, there was notable tension between understanding that street tree management actions could affect specific services or disservices, and whether and to what degree decisions should, or could, be aligned with a specific benefit, and beneficiaries thereof.

*“P2: I think if we’re gonna sell it to folks, maybe public health is the way to do it. Because if you basically say, like, listen, your grandchild is going to have asthma. Do you want that to happen? Most people, I would like to think, would say no, right? So, how about we plant a bunch of trees and take care of the existing ones that we have?*

*Interviewer: Do you think that would, then, make you choose species with less allergens?*

*P2: Yeah. I guess that’s a good point. There’s a lot of checks and balances there.”*

When it came to aligning street tree management decisions with a specific benefit, many said explicitly that ecosystem services were not considered directly in their typical decision-making processes (n = 10). However, they did acknowledge that choices they make could affect alignment with a specific benefit goal.

*“I don’t go out every day, looking at ‘Oh, how can I sequester this carbon longer?’ That’s, I mean, it’s a mutual, happy, coincidental benefit that comes along with all of the tangible everyday benefits that the residents get that they don’t think about.” (P13)*

“*[Our i-Tree ecosystem service assessment] is not really for management decisions. That’s more for budgeting, public information. I don’t think we use it when deciding where to plant a tree or which tree to plant.” (P6)*

When asked to compare the importance of a street tree management objective related to increasing canopy or tree health with an ecosystem service goal they previously described as very important to their city, most stated that their street tree objective was more important, but still believed it would align with city ecosystem service goals, at least broadly.

*“Oh, [the more important objective would] be planting within our neighborhoods just because the byproduct of doing that will help to mitigate stormwater.” (P11)*

However, even if their overall program goals were focused on metrics of urban forest structure such as canopy cover, many also could describe situations where ecosystem services were used to direct management choices (n = 14). These were mostly related to managing sites with specific needs, such as sidewalks needing shade for pedestrians, or a riparian area in need of native species for local wildlife. At the city scale, ecosystem services were used to direct management choices usually only at the stage of prioritizing new plantings within certain neighborhoods with specific needs.

*“I’ve highlighted the site suitability for different metrics. So, you know, living below poverty level, high urban heat island effect, low tree canopy, looking at the health index, [...] and pulled those census blocks out [...] So that map, I’m actually including in the RFP, and asking the consultant to specifically identify potential tree planting sites in those census blocks.” (P17)*

#### Role of street trees in city ecosystem service goals

When asked about the role of street trees in specific plans in their cities, participants described important roles (n = 8), such as playing key parts in specific strategies, to minor roles (n = 4), like being mentioned but not receiving sufficient attention, to not being mentioned at all (n = 2).

*“I’ve been involved with the development of that plan. It certainly discusses trees, but I don’t think there’s specific targets for trees within the [Vision Zero plan to eliminate traffic-related mortalities]. Just some narrative around, you know, the benefits of having a nice green streetscape, reducing traffic speeds and things like that.” (P18)*

However, some expressed dismay over arbitrary tree-related objectives being set that were unrealistic or not evidence-based, or not supported by city practices (n = 6).

*“...I just feel like it’s lip service at its finest, because, you know, all we’re really doing is counting how many trees we’re planting. And you know, that really isn’t a great proxy for anything at all because, you know, half of them are gonna die anyways, and we don’t really know how it’s advancing our goals, right?” (P16)*

#### Role of city ecosystem service goals in street tree management

Despite sharing a range of perceptions on the role of street trees in their city’s sustainability plans, participants frequently described the ecosystem service goals within these municipal sustainability plans as having a small influence on their decisions (n = 7). Ecosystem services were either not related to their day-to-day operations, or many competing priorities prevented them from adhering to one plan or another. The type of plan, the legal authority of that plan, and the funding allocated by that plan influenced how much it affected their management decisions.

*“...We do refer to the Climate Action Plan, [...] but overall, like, from a day-to-day standpoint, it’s just trying to get more trees in more spaces.” (P20)*

The term “guide” recurred frequently in reference to how they viewed municipal sustainability plans, such as climate action plans, especially in describing them as a flexible guide to follow and when useful or practical for their own urban forestry goals (n = 7).

*“...I don’t use them as my Bible [...] my focus is health of trees, health of the urban forest, you know, keeping what we have as long as we can [...] I think the benefit of that is, is providing what we need for those goals [...] my goal is the forest, their goal is they want these things to happen. But I just use that as like guides.” (P20)*

Participants also viewed city ecosystem service goals, and the plans that contained them, as having a role in garnering recognition, political and public support, and funding, for their programs (n = 8).

*“I mean, sure, that goes in any grant application I’m working on. It definitely comes into play, like, I got funding from a different department to create this assistant position from water quality.” (P7)*

*“I’m hoping that through the Climate Change Action Plan review we can maybe find opportunities for more funding for maintaining our forest.” (P8)*

## Discussion

Urban foresters rarely aligned their street tree management decisions with adopted, specific, ecosystem service goals in their municipalities. What, then, guides their decision-making? And how will these decisions impact sustainability goals? Below, we discuss how challenges urban foresters faced in aligning their programs with specific city-level goals were eased by following a widely adopted “more, bigger trees” paradigm. We have synthesized literature which supports how this approach may align with some city priorities—and how this tension gets to the heart of the purpose of urban forestry.

### Extent of influence of adopted municipal ecosystem service goals on site-level decision-making

Given how pervasive and widespread the concept of ecosystem services is in urban forestry (Roman et al. 2021; Ordóñez Barona et al. 2024b), one might expect to see that practice would embrace sustainability goals framed as ecosystem services and forsake more holistic ecosystems perspectives—fulfilling the fears that the concept could blind the complexity of ecosystems (Norgaard 2010). We found that although participants could describe cases where their decision-making was intentionally aligned with a particular ecosystem service goal, their programs were typically aligned with achieving management objectives that centered on trees or ecosystem integrity, such as increasing tree cover, reducing risk of tree failure, or even restoring historical ecosystem structures. Notably, the tree risk objective is oriented around minimizing disservices, rather than increasing services per se. Participants who believed their programs were aligned with ecosystem service goals often thought this because they believed their tree-centered ecosystem structure goals (to increase the abundance and health of their urban forests in general) would necessarily contribute to the ecosystem service goals in their city’s plans.

The finding that urban foresters typically prioritize tree-centered goals over ecosystem service goals provides critical context to understanding participants’ views on aligning their programs with so-called “other” city goals. While urban foresters agreed that urban forests provide many diverse ecosystem services, and believed that their responsibilities include increasing such provision, any work to tailor programs toward specific ecosystem service goals was often perceived as “extra” or “additional” to the core function of their jobs. It is significant that the mainstreaming of rhetoric around ecosystem services in urban forestry (Silvera Seamans 2013; Young 2013) has not resulted in the widespread uptake of using specific ecosystem services to direct management and operations. This is true despite evidence of the integration of urban trees into municipal plans as strategies for specific goals (Supplement; Table S3). Even though our sample only included cities that had adopted ecosystem service goals, and we do not have direct evidence on other cities, we think it is reasonable based on past literature (e.g., Ordóñez Barona et al. 2024b) to expect that urban foresters in cities in the US and Canada without adopted ecosystem service goals also prioritize tree-centered goals over goals framed as ecosystem services.

While the overall concept of ecosystem services is simple—that ecosystems, such as urban forests, provide benefits to human well-being—the challenges to using or operationalizing this concept can range from the conceptual to the technical to even the ethical (Luck et al. 2012; Carmen et al. 2018). Interestingly, we found participants to be remarkably united around a shared understanding that urban forests provided a broad swath of essential benefits, and that increasing tree numbers, tree health, and canopy cover would necessarily result in more of such benefits. In contrast to challenges that other environmental fields have encountered in agreeing upon methods to assess ecosystem services (Jax et al. 2018; Ainscough et al. 2019), there was also no disagreement on how to assess ecosystem services—if discussed, most used i-Tree, or tools using i-Tree models.

When it came to operationalizing ecosystem services in a more technical, targeted way, such as through aligning decisions with particular ecosystem service goals, participants seemed less practiced at discussing management in such a way, with the notable exception of using spatial indicators of ecosystem services need to direct tree planting. This mirrored evidence from a large European survey (Ainscough et al. 2019) finding that environmental management practitioners, broadly, do not see ecosystem services as useful in supporting decision-making, in sharp contrast to the perception of the usefulness of the concept among ecosystem services researchers. Similar to findings of studies of urban foresters in Australia and the UK (e.g., Davies et al. 2017; Roy et al. 2017), our participants seemed to use the language and metrics of ecosystem services primarily to advocate for their current programs and only opportunistically to guide their site-level decisions, especially if mainstreamed in their cities and provided opportunities to access funding and other support. However, overall, they do not believe their management exists to serve one particular service or another—as evidenced by the cases in our results where participants resisted the idea of alignment with single services—and are instead guided by a sense of the greater purpose of urban forestry to provide benefits to the public they serve.

### Best strategies and current challenges in aligning site-level decisions to contribute to specific ecosystem service goals

Despite the potential pitfalls related to adopting ecosystem services framing in municipal planning, the sustainability goals cities are adopting like urban heat mitigation, stormwater management, and many more, still represent critical challenges to health and well-being and provide needed specificity (e.g. Biddle and Koontz 2014) around which programs could be oriented. From a technical perspective, our analysis indicated that governance factors, such as staffing, insufficient funding, and departmental silos, were the primary challenges to municipal urban foresters faced in alignment with the ambitious ecosystem service goals cities are setting (See Results, sub-section "Challenges to align street tree management with specific city ecosystem service goals"). Even their institutional arrangement (e.g., their department) or structure of their current operational procedures (e.g., being service request-based in some instances) were barriers to strategically aligning with specific ecosystem service goals. These challenges are mostly expected as they have been identified as barriers to program improvement by previous research (Davies et al. 2017; Ordóñez et al. 2020), and it is difficult to parse challenges to tree survival from those that influence the benefits of those same trees. Problems with insufficient funding and staffing make it yet more imperative for cities to use wisely what resources do exist, and to align spending with the most critical goals.

Our results nonetheless support the research showing that urban forest management can be tailored to advance specific goals, and that urban foresters leading advanced programs in the US and Canada have specialized knowledge and access to a field that is rapidly advancing in professionalism (O’Herrin et al. 2023). However, for cities attempting to address challenges through setting ecosystem service goals, in the absence of strategies to facilitate alignment with a particular ecosystem service goal, such as allocated funding and collaborative partnerships, our results indicate that urban foresters such as those we interviewed prioritize managing for public safety and a “more, bigger trees” approach. Our participants used this approach to make the implementation of their programs relevant to policy goals, or at least demonstrate existing alignment of their urban forestry programs with ecosystem service goals from other fields.

### Guided by “more, bigger trees” or ecosystem service goals?

Our results show that the way professionals understand mechanisms of ecosystem service provision can affect their decision-making. In the case of urban foresters in the US and Canada, we found the widespread belief that “more, bigger trees” would virtually always lead to more ecosystem services, in a general sense, and working toward urban forestry objectives which aligned with this would necessarily contribute to most of the goals in their cities’ sustainability agendas. This paradigm is rooted in ideas such as the “large tree argument,” which posits that large stature trees should be prioritized because, in many cases, they offer more per tree benefits to society (USDA Forest Service 2004). The broader “Trees are Good” campaign, promoted by the arboricultural industry (Roman et al. 2021), also follows the logic of the “more, bigger trees” paradigm through its promotion of information on the benefits of trees to people to encourage proper tree care. Management paradigms like these, while never perfect, can be important and useful heuristics to prevent decision-making from being paralyzed by the infinite complexity of the real world (Levine et al. 2015). The prevalence of the “more, bigger trees” paradigm suggests it is an example of the adoption of a group-based value by a profession (Breed 2022), in this case, among urban foresters in the US and Canada. This paradigm may give professionals a shared mission as well as facilitate their translation of tree-centered objectives from the city scale, like setting canopy cover goals, to the site-scale, like planting larger specimens wherever possible.

Striving for “more, bigger trees” does align with many critical sustainability goals that cities are setting. Larger stature trees provide a greater cooling effect (Rahman et al. 2020), intercept more stormwater (Dowtin et al. 2023), sequester and store more carbon (Nowak and Crane 2002), provide critical habitat to urban wildlife (Stagoll et al. 2012), and even serve as unique historical and cultural attachment points (Lavy and Zavar 2023). However, solely following a “more, bigger trees” approach misses additional factors needed for all these goals, such as considering species traits like crown structure and density, leaf texture, or native status (Castro-Díez et al. 2019; Farrell et al. 2022; Dowtin et al. 2023).

One benefit where simply managing for “more trees” without place-based nuance may exacerbate disservices, is with respect to air quality, a frequently promoted benefit. Streets with a canyon-like form can create worse air quality conditions when they are fully planted with tall and wide canopied trees as the trees can decrease air flow and trap pollutants (Eisenman et al. 2019); in this context, fewer trees with increased spacing can mitigate this negative effect (Abhijith et al. 2017). Another often-touted benefit is that more trees can make cities safer. However, dense trees can also affect fear of crime (Jansson et al. 2013), and have been associated with facilitating crime, because they can obstruct lines of sight (though primarily with vegetation at eye-level, while presence of taller trees is associated with reduced crime rates) (Lin et al. 2021). Similarly, a focus on only “bigger trees” misses unique benefits provided by smaller stature species. Many trees valued for fruit and flowers tend to be small stature, and are highly demanded by residents for food production, cultural value, and aesthetic appeal (Nguyen et al. 2017). Small stature trees can also provide significant benefits which align with city ecosystem service goals while accommodating space constraints from built infrastructure (e.g., utilities). Indeed, street tree systems in some US cities have been shifting away from large stature trees to small stature trees to avoid the ecosystem disservices associated with tree-utility conflicts (Magarik et al. 2020; Roman and Eisenman 2022). The “more, bigger trees” paradigm may align with many critical sustainability goals, but it is a somewhat blunt instrument to apply in all situations, especially those needing a more nuanced consideration of desired benefits and the actual beneficiaries, as well as site-specific tradeoffs across different services, or between services and disservices.

Urban foresters across the US and Canada are guided, and united, by their own sense of the purpose of urban forestry to grow healthy forests which serve urban communities, which mostly aligns but is in some cases in contradiction with the objectives set by city leaders. Based upon the results of our interviews with members of this group, we posit that their perception of the purpose of urban forestry is mediated by the development of the professional identity of urban foresters around shared values (Breed 2022; O’Herrin et al. 2023) and grounded in management paradigms backed by the urban forestry scientific community (USDA Forest Service 2004). In our interviews with urban foresters leading advanced programs, from small to large cities, we find that ecosystem services as a concept (and accompanying metrics and language) is more used to gain support for their programs and justify all management in line with the “more, bigger trees” paradigm, than to enable alignment of site-level management actions with a specific sustainability goal. However, we did find cases where factors like dedicated funding sources, evidence-backed decision support tools, and interdisciplinary collaboration aligned site-level decisions toward specific goals, especially urban heat reduction and stormwater mitigation (see Results, sub-section "Strategies to align street tree management with specific city ecosystem service goals").

### Limitations

Our interviews did not focus on a specific type of planning document or specific type of ecosystem service goal. Different plans are imbued with varying authority depending on the governance context of a municipality. Additionally, qualitative research such as this is best suited for suggesting the potential spread of ideas and for developing new hypotheses. Future research on the alignment of urban forest management decision-making could reveal advantages of utilizing different administrative mechanisms over others, as well as investigate the degree of genuine collaboration and communication across municipal departments in the planning process. Because we sought to draw general conclusions across a profession, the results are also limited by small sample sizes for specific regions, for example, we were unable to recruit participants from the Canadian prairies or maritime provinces. The results are most relevant in the US and Canada, as it has been recognized that the role of urban foresters employed by municipalities takes very different forms around the world (Yao et al. 2019; Ordóñez Barona et al. 2023). Study results should be compared with other social and ecological contexts, rather than assumed to be representative. However, there are parallels to be drawn at a wider scale as the concept of ecosystem services, and demand for tools, certainly impacts the research and practice of urban forestry around the world (Wu et al. 2019; Riondato et al. 2020; Xie et al. 2023).

## Conclusions

The sustainability goals that cities are working toward are not trivial; rather, they will significantly impact the quality of life of urban residents, mortality rates and the burdens of disease. So far, how cities are setting sustainability goals and working to align their many programs, including nature-based solutions like urban forestry, appears to influence urban foresters’ actions only modestly, inspiring them to align decision-making with specific goals opportunistically rather than as an organizing principle for their whole programs. By pursuing the “more, bigger trees” paradigm, urban forest professionals are likely making decisions in alignment with many city goals, while potentially also missing opportunities or causing unintended disservices. In the context of increasing alignment with adopted goals, urban foresters expressed a desire for new tools and evidence to support better alignment with ecosystem service goals, as well as more collaboration with other professionals like urban planners, stormwater managers, etc. Overall, to be an urban forester is to constantly be “the voice for trees” in the endlessly complex and changing ecosystems of cities. In the face of this complexity, we posit that professionals implementing and caring for nature-based solutions are guided by a shared sense of the immense values provided by nature to cities, which usually aligns but sometimes conflicts with specific city goals. Urban planners and city leaders can use these results to understand the barriers to scaling site-level actions to strategic goals, and the knowledge and motivations of the fields they need to unite in serve of such goals.

## Supplementary Information

Below is the link to the electronic supplementary material.

Supplementary file1 (PDF 250 KB)

## Data Availability

Anonymous exemplar quotes from interviews and other participant data are included in the body of the manuscript. Additional data will not be publicly available because they contain information that could compromise the privacy of research participants and because participants did not consent for these to be made public. Ethical approval for this research study was granted by the Behavioural Research Ethics Board of the University of British Columbia (Approval Certificate #H22-02611-A002).

## References

[CR1] Abhijith, K. V., P. Kumar, J. Gallagher, A. McNabola, R. Baldauf, F. Pilla, B. Broderick, S. Di Sabatino, and B. Pulvirenti. 2017. Air pollution abatement performances of green infrastructure in open road and built-up street canyon environments – A review. *Atmospheric Environment* 162: 71–86.

[CR2] Abson, D. J., H. von Wehrden, S. Baumgärtner, J. Fischer, J. Hanspach, W. Härdtle, H. Heinrichs, A. M. Klein, et al. 2014. Ecosystem services as a boundary object for sustainability. *Ecological Economics* 103: 29–37.

[CR3] Ainscough, J., A. de Vries Lentsch, M. Metzger, M. Rounsevell, M. Schröter, B. Delbaere, R. de Groot, and J. Staes. 2019. Navigating pluralism: Understanding perceptions of the ecosystem services concept. *Ecosystem Services* 36: 100892.

[CR35] American Forests. 2024. Tree Equity Score. https://www.treeequityscore.org/.

[CR4] Angelo, H., J. Sirigotis, and K. MacFarlane. 2020. *The Challenge of Equity in California’s Municipal Climate Action Plans*.

[CR5] Arbor Day Foundation. 2024a. Tree City USA. https://www.arborday.org/programs/treecityusa/.

[CR6] Arbor Day Foundation. 2024b. Tree Cities of the World. https://treecitiesoftheworld.org/.

[CR7] Arkema, K. K., G. M. Verutes, S. A. Wood, C. Clarke-Samuels, S. Rosado, M. Canto, A. Rosenthal, M. Ruckelshaus, et al. 2015. Embedding ecosystem services in coastal planning leads to better outcomes for people and nature. *Proceedings of the National Academy of Sciences* 112: 7390–7395. 10.1073/pnas.1406483112 PMC4475972 26082545

[CR9] Barron, S., M. Egerer, A. Almas, J. Rayner, D. Bell, R. Hauer, C. Konijnendijk, E. Kwun, et al. 2025. Taking Stock: The Current State of Urban Forestry Education at International Institutions of Higher Education. *Arboriculture & Urban Forestry (AUF)*.

[CR10] Bassett, C. G., S. D. Day, C. C. Konijnendijk, L. A. Roman, and V. Hemming. 2024. Aligning urban forest management actions with urban sustainability goals: A multi-city expert elicitation. *Ecosphere* 15: e70120.

[CR11] Biddle, J. C., and T. M. Koontz. 2014. Goal specificity: A proxy measure for improvements in environmental outcomes in collaborative governance. *Journal of Environmental Management* 145: 268–276. 25083592 10.1016/j.jenvman.2014.06.029

[CR12] Bodnaruk, E. W., C. N. Kroll, Y. Yang, S. Hirabayashi, D. J. Nowak, and T. A. Endreny. 2017. Where to plant urban trees? A spatially explicit methodology to explore ecosystem service tradeoffs. *Landscape and Urban Planning* 157: 457–467.

[CR13] Breed, C. A. 2022. Value negotiation and professional self-regulation – Environmental concern in the design of the built environment. *Urban Forestry & Urban Greening* 74: 127626.

[CR14] Bruner, S. G., M. I. Palmer, K. L. Griffin, and S. Naeem. 2023. Planting design influences green infrastructure performance: Plant species identity and complementarity in rain gardens. *Ecological Applications* 33: e2902. 37345972 10.1002/eap.2902

[CR15] Carmen, E., A. Watt, L. Carvalho, J. Dick, I. Fazey, G. Garcia-Blanco, B. Grizzetti, J. Hauck, et al. 2018. Knowledge needs for the operationalisation of the concept of ecosystem services. *Ecosystem Services* 29: 441–451.

[CR16] Castro-Díez, P., A. S. Vaz, J. S. Silva, M. van Loo, Á. Alonso, C. Aponte, Á. Bayón, P. J. Bellingham, et al. 2019. Global effects of non-native tree species on multiple ecosystem services. *Biological Reviews* 94: 1477–1501. 30974048 10.1111/brv.12511 PMC6850375

[CR17] Chan, K. M. A., M. R. Shaw, D. R. Cameron, E. C. Underwood, and G. C. Daily. 2006. Conservation Planning for Ecosystem Services. *PLOS Biology* 4: e379. 17076586 10.1371/journal.pbio.0040379 PMC1629036

[CR18] Chan, K. M. A., T. Satterfield, and J. Goldstein. 2012. Rethinking ecosystem services to better address and navigate cultural values. *Ecological Economics* 74: 8–18.

[CR19] Chan, K. M. A., R. K. Gould, and U. Pascual. 2018. Editorial overview: Relational values: What are they, and what’s the fuss about? *Current Opinion in Environmental Sustainability* 35: A1–A7.

[CR20] Cheng, Z., S. Nitoslawski, C. Konijnendijk van den Bosch, S. Sheppard, L. Nesbitt, and C. Girling. 2021. Alignment of municipal climate change and urban forestry policies: A Canadian perspective. *Environmental Science & Policy* 122: 14–24.

[CR21] Cortinovis, C., and D. Geneletti. 2019. A framework to explore the effects of urban planning decisions on regulating ecosystem services in cities. *Ecosystem Services* 38: 100946.

[CR22] Daily, G. C. 1997. *Nature’s services*. Washington, DC: Island Press.

[CR23] Daily, G. C., S. Polasky, J. Goldstein, P. M. Kareiva, H. A. Mooney, L. Pejchar, T. H. Ricketts, J. Salzman, et al. 2009. Ecosystem services in decision making: Time to deliver. *Frontiers in Ecology and the Environment* 7: 21–28.

[CR24] Daniel, T. C., A. Muhar, A. Arnberger, O. Aznar, J. W. Boyd, K. M. A. Chan, R. Costanza, T. Elmqvist, et al. 2012. Contributions of cultural services to the ecosystem services agenda. *Proceedings of the National Academy of Sciences* 109: 8812–8819. 10.1073/pnas.1114773109 PMC3384142 22615401

[CR25] Davies, H. J., K. J. Doick, M. D. Hudson, and K. Schreckenberg. 2017. Challenges for tree officers to enhance the provision of regulating ecosystem services from urban forests. *Environmental Research* 156: 97–107. 28342350 10.1016/j.envres.2017.03.020

[CR26] Day, S. D., P. Ries, C. G. Bassett, P. E. Wiseman, and K. O’Herrin. 2022. Support for a new credential in urban forestry: Results from a survey of urban forest professionals. *Urban Forestry & Urban Greening* 73: 127588.

[CR93] Davey Tree. 2024. TreeKeeper®. https://www.davey.com/environmental-consulting-services/treekeeper-inventory-management-software/.

[CR27] Dickie, I. A., B. M. Bennett, L. E. Burrows, M. A. Nuñez, D. A. Peltzer, A. Porté, D. M. Richardson, M. Rejmánek, et al. 2014. Conflicting values: Ecosystem services and invasive tree management. *Biological Invasions* 16: 705–719.

[CR28] Döringer, S. 2021. ‘The problem-centred expert interview’. Combining qualitative interviewing approaches for investigating implicit expert knowledge. *International Journal of Social Research Methodology* 24: 265–278.

[CR29] Dorst, H., A. van der Jagt, R. Raven, and H. Runhaar. 2019. Urban greening through nature-based solutions – Key characteristics of an emerging concept. *Sustainable Cities and Society* 49: 101620.

[CR30] Dowtin, A. L., B. C. Cregg, D. J. Nowak, and D. F. Levia. 2023. Towards optimized runoff reduction by urban tree cover: A review of key physical tree traits, site conditions, and management strategies. *Landscape and Urban Planning* 239: 104849.

[CR31] Eisenman, T. S., G. Churkina, S. P. Jariwala, P. Kumar, G. S. Lovasi, D. E. Pataki, K. R. Weinberger, and T. H. Whitlow. 2019. Urban trees, air quality, and asthma: An interdisciplinary review. *Landscape and Urban Planning* 187: 47–59.

[CR32] Escobedo, F. J., V. Giannico, C. Y. Jim, G. Sanesi, and R. Lafortezza. 2019. Urban forests, ecosystem services, green infrastructure and nature-based solutions: Nexus or evolving metaphors? *Urban Forestry & Urban Greening* 37.

[CR33] Farrell, C., S. J. Livesley, S. K. Arndt, L. Beaumont, H. Burley, D. Ellsworth, M. Esperon-Rodriguez, T. D. Fletcher, et al. 2022. Can we integrate ecological approaches to improve plant selection for green infrastructure? *Urban Forestry & Urban Greening* 76: 127732.

[CR34] Ferrini, F., C. C. K. van den Bosch, A. Fini, and Taylor & Francis eBooks A-Z, editors. 2017. *Routledge handbook of urban forestry*. Routledge, London; New York.

[CR36] Frantzeskaki, N. 2019. Seven lessons for planning nature-based solutions in cities. *Environmental Science & Policy* 93: 101–111.

[CR37] Gómez-Baggethun, E., and D. N. Barton. 2013. Classifying and valuing ecosystem services for urban planning. *Ecological Economics* 86: 235–245.

[CR38] Grant, A., A. A. Millward, S. Edge, L. A. Roman, and C. Teelucksingh. 2022. Where is environmental justice? A review of US urban forest management plans. *Urban Forestry & Urban Greening* 77: 127737.

[CR39] Guest, G., A. Bunce, and L. Johnson. 2006. How Many Interviews Are Enough?: An Experiment with Data Saturation and Variability. *Field Methods* 18: 59–82.

[CR40] Hansen, R., and S. Pauleit. 2014. From Multifunctionality to Multiple Ecosystem Services? A Conceptual Framework for Multifunctionality in Green Infrastructure Planning for Urban Areas. *Ambio* 43: 516–529 10.1007/s13280-014-0510-2. 24740622 10.1007/s13280-014-0510-2 PMC3989511

[CR41] Hauer, R. J., and W. D. Peterson. 2016. *Municipal Tree Care and Management in the United States: A 2014 Urban & Community Forestry Census of Tree Activities*. Page 71. Special Publication, College of Natural Resources, University of Wisconsin – Stevens Point.

[CR42] Huff, E. S., M. L. Johnson, L. A. Roman, N. F. Sonti, C. C. Pregitzer, L. K. Campbell, and H. McMillen. 2020. A Literature Review of Resilience in Urban Forestry. *Arboriculture & Urban Forestry* 46.

[CR43] Jackson, L. E., J. Daniel, B. McCorkle, A. Sears, and K. F. Bush. 2013. Linking ecosystem services and human health: The Eco-Health Relationship Browser. *International Journal of Public Health* 58: 747–755. 23877533 10.1007/s00038-013-0482-1

[CR44] Jansson, M., H. Fors, T. Lindgren, and B. Wiström. 2013. Perceived personal safety in relation to urban woodland vegetation – A review. *Urban Forestry & Urban Greening* 12: 127–133.

[CR45] Jax, K., E. Furman, H. Saarikoski, D. N. Barton, B. Delbaere, J. Dick, G. Duke, C. Görg, et al. 2018. Handling a messy world: Lessons learned when trying to make the ecosystem services concept operational. *Ecosystem Services* 29: 415–427.

[CR46] Keith, L., C. J. Gabbe, and E. Schmidt. 2023. Urban heat governance: Examining the role of urban planning. *Journal of Environmental Policy & Planning* 25: 642–662.

[CR47] Klain, S. C., T. A. Satterfield, and K. M. A. Chan. 2014. What matters and why? Ecosystem services and their bundled qualities. *Ecological Economics* 107: 310–320.

[CR48] Koeser, A. K., R. J. Hauer, J. W. Miesbauer, and W. Peterson. 2016. Municipal tree risk assessment in the United States: Findings from a comprehensive survey of urban forest management. *Arboricultural Journal* 38: 218–229.

[CR49] Lam, S. T., and T. M. Conway. 2018. Ecosystem services in urban land use planning policies: A case study of Ontario municipalities. *Land Use Policy* 77: 641–651.

[CR50] Lavy, B. L., and E. Zavar. 2023. Recovering the urban forest: The role of trees, tree culture, and place attachment before and after Hurricane Harvey. *Urban Forestry & Urban Greening*: 127949.

[CR51] Levine, J., K. M. A. Chan, and T. Satterfield. 2015. From rational actor to efficient complexity manager: Exorcising the ghost of *Homo economicus* with a unified synthesis of cognition research. *Ecological Economics* 114: 22–32.

[CR52] Liang, S., and Y. Fu. 2023, July 1. Otter.ai. Otter.ai, Inc., Mountain View, California, USA.

[CR53] Lilly, S. J., C. G. Bassett, J. Komen, and L. Purcell. 2022. *Arborists’ Certification Study Guide*, 4th ed. Atlanta, GA United States: Fourth edition. International Society of Arboriculture.

[CR54] Lin, J., Q. Wang, and B. Huang. 2021. Street trees and crime: What characteristics of trees and streetscapes matter. *Urban Forestry & Urban Greening* 65: 127366.

[CR55] Luck, G. W., K. M. A. Chan, U. Eser, E. Gómez-Baggethun, B. Matzdorf, B. Norton, and M. B. Potschin. 2012. Ethical considerations in on-ground applications of the ecosystem services concept. *BioScience* 62: 1020–1029.

[CR56] Magarik, Y. A. S., L. A. Roman, and J. G. Henning. 2020. How should we measure the DBH of multi-stemmed urban trees? *Urban Forestry & Urban Greening* 47: 126481.

[CR57] Matzek, V., and K. A. Wilson. 2021. Public support for restoration: Does including ecosystem services as a goal engage a different set of values and attitudes than biodiversity protection alone? *PLoS ONE* 16: e0245074. 33465097 10.1371/journal.pone.0245074 PMC7815106

[CR58] McPherson, E. G., A. Kendall, and S. Albers. 2015. Life cycle assessment of carbon dioxide for different arboricultural practices in Los Angeles. *CA. Urban Forestry & Urban Greening* 14: 388–397.

[CR59] Miller, R. W., R. J. Hauer, and L. P. Werner. 2015. *Urban forestry: Planning and management of urban greenspaces*. Long Grove: Waveland.

[CR8] Millennium Ecosystem Assessment, editor. 2005. *Ecosystems and human well-being: synthesis*. Island Press, Washington, DC.

[CR60] Naidoo, R., and T. H. Ricketts. 2006. Mapping the Economic Costs and Benefits of Conservation. *PLOS Biology* 4: e360. 17076583 10.1371/journal.pbio.0040360 PMC1629040

[CR61] Nelson, E., G. Mendoza, J. Regetz, S. Polasky, H. Tallis, Dr. Cameron, K. M. Chan, G. C. Daily, et al. 2009. Modeling multiple ecosystem services, biodiversity conservation, commodity production, and tradeoffs at landscape scales. *Frontiers in Ecology and the Environment* 7: 4–11.

[CR62] Nguyen, V. D., L. A. Roman, D. H. Locke, S. K. Mincey, J. R. Sanders, E. Smith Fichman, M. Duran-Mitchell, and S. L. Tobing. 2017. Branching out to residential lands: Missions and strategies of five tree distribution programs in the U.S. *Urban Forestry & Urban Greening* 22: 24–35.

[CR63] Norgaard, R. B. 2010. Ecosystem services: From eye-opening metaphor to complexity blinder. *Ecological Economics* 69: 1219–1227.

[CR64] Nowak, D. J., and D. E. Crane. 2002. Carbon storage and sequestration by urban trees in the USA. *Environmental Pollution* 116: 381–389. 11822716 10.1016/s0269-7491(01)00214-7

[CR65] Nowak, D. J. 2020. Understanding i-Tree: summary of programs and methods. *General Technical Report NRS-200. Madison, WI: U.S. Department of Agriculture, Forest Service, Northern Research Station. 100 p.* 200: 1–100.

[CR66] NVivo. 2023. Lumivero.

[CR67] O’Herrin, K., P. E. Wiseman, S. D. Day, and R. J. Hauer. 2020. Professional identity of urban foresters in the United States. *Urban Forestry & Urban Greening* 54: 126741.

[CR68] O’Herrin, K., C. G. Bassett, S. D. Day, P. D. Ries, and P. E. Wiseman. 2023. Borrowed Credentials and Surrogate Professional Societies: A Critical Analysis of the Urban Forestry Profession. *Arboriculture & Urban Forestry* 49: 107–136.

[CR69] Olander, L. P., R. J. Johnston, H. Tallis, J. Kagan, L. A. Maguire, S. Polasky, D. Urban, J. Boyd, et al. 2018. Benefit relevant indicators: Ecosystem services measures that link ecological and social outcomes. *Ecological Indicators* 85: 1262–1272.

[CR70] Ordóñez, C., C. G. Threlfall, D. Kendal, D. F. Hochuli, M. Davern, R. A. Fuller, R. van der Ree, and S. J. Livesley. 2019. Urban forest governance and decision-making: A systematic review and synthesis of the perspectives of municipal managers. *Landscape and Urban Planning* 189: 166–180.

[CR71] Ordóñez, C., C. G. Threlfall, S. J. Livesley, D. Kendal, R. A. Fuller, M. Davern, R. van der Ree, and D. F. Hochuli. 2020. Decision-making of municipal urban forest managers through the lens of governance. *Environmental Science & Policy* 104: 136–147.

[CR72] Ordóñez Barona, C., A. A. Eleuterio, A. Vasquez, T. Devisscher, M. D. Baptista, C. Dobbs, L. Orozco-Aguilar, and E. Meléndez-Ackerman. 2023. Views of government and non-government actors on urban forest management and governance in ten Latin-American capital cities. *Land Use Policy* 129: 106635.

[CR73] Ordóñez Barona, C., A. Jain, M. Heppner, A. St Denis, D. Boyer, J. Lane, C. Edwards, P. Duinker, et al. 2024a. Gaps in the implementation of urban forest management plans across canadian cities. *Landscape and Urban Planning* 251: 105168.

[CR74] Ordóñez Barona, C., A. St Denis, J. Jung, C. G. Bassett, S. Delagrange, P. Duinker, and T. Conway. 2024b. A content analysis of urban forest management plans in Canada: Changes in social-ecological objectives over time. *Landscape and Urban Planning* 251: 105154.

[CR75] Polasky, R. de G., B. Fisher, M. Christie, J. Aronson, L. Braat, J. Gowdy, R. Haines-Young, E. Maltby, A. Neuville, 2011. Integrating the Ecological and Economic Dimensions in Biodiversity and Ecosystem Service Valuation. Page *The Economics of Ecosystems and Biodiversity: Ecological and Economic Foundations*. Routledge.

[CR76] Potschin, M. B., and R. H. Haines-Young. 2011. Ecosystem services: Exploring a geographical perspective. *Progress in Physical Geography: Earth and Environment* 35: 575–594.

[CR77] Powning, C. B., R. W. Harper, D. V. Bloniarz, K. J. Kahl, and E. M. Markowitz. 2024. Reviewing the use of research interviews and qualitative inquiry in urban forestry: Understanding human-tree relationships in the built landscape. *Urban Forestry & Urban Greening* 98: 128387.

[CR78] Quinton, J., L. Nesbitt, D. Sax, and L. Harris. 2024. Greening the gentrification process: Insights and engagements from practitioners. *Environment and Planning E: Nature and Space*:25148486241236281. 10.1177/25148486241236281 PMC13032840 41909531

[CR79] Rahman, M. A., L. M. F. Stratopoulos, A. Moser-Reischl, T. Zölch, K.-H. Häberle, T. Rötzer, H. Pretzsch, and S. Pauleit. 2020. Traits of trees for cooling urban heat islands: A meta-analysis. *Building and Environment* 170: 106606.

[CR80] Riondato, E., F. Pilla, A. Sarkar Basu, and B. Basu. 2020. Investigating the effect of trees on urban quality in Dublin by combining air monitoring with i-Tree Eco model. *Sustainable Cities and Society* 61: 102356.

[CR81] Roman, L. A., and T. S. Eisenman. 2022. *Drivers of street tree species selection: The case of London planetrees in Philadelphia*. Page The Politics of Street Trees: Routledge.

[CR82] Roman, L. A., T. M. Conway, T. S. Eisenman, A. K. Koeser, C. Ordóñez Barona, D. H. Locke, G. D. Jenerette, J. Östberg, et al. 2021. Beyond ‘trees are good’: Disservices, management costs, and tradeoffs in urban forestry. *Ambio* 50: 615–630 10.1007/s13280-020-01396-8. 33011917 10.1007/s13280-020-01396-8 PMC7882647

[CR83] Roy, S., A. Davison, and J. Östberg. 2017. Pragmatic factors outweigh ecosystem service goals in street tree selection and planting in South-East Queensland cities. *Urban Forestry & Urban Greening* 21: 166–174.

[CR84] Sax, D., C. Manson, and L. Nesbitt. 2020. Governing for Diversity: An Exploration of Practitioners’ Urban Forest Preferences and Implications for Equitable Governance. *Frontiers in Sustainable Cities* 2.

[CR85] Silvera Seamans, G. 2013. Mainstreaming the environmental benefits of street trees. *Urban Forestry & Urban Greening* 12: 2–11.

[CR86] Society of American Foresters. 2024. *SAF Certified Urban & Community Forester Exam Domains and Knowledge Areas*. Page 7.

[CR87] Srivastava, P., and N. Hopwood. 2009. A Practical Iterative Framework for Qualitative Data Analysis. *International Journal of Qualitative Methods* 8: 76–84.

[CR88] Stagoll, K., D. B. Lindenmayer, E. Knight, J. Fischer, and A. D. Manning. 2012. Large trees are keystone structures in urban parks. *Conservation Letters* 5: 115–122.

[CR89] Stone, B., J. Vargo, and D. Habeeb. 2012. Managing climate change in cities: Will climate action plans work? *Landscape and Urban Planning* 107: 263–271.

[CR90] Thompson, K., K. Sherren, and P. N. Duinker. 2019. The use of ecosystem services concepts in Canadian municipal plans. *Ecosystem Services* 38: 100950.

[CR91] Thompson, K., K. Sherren, P. N. Duinker, M. Terashima, and A. Hayden. 2024. Building the case for protecting urban nature: How urban planners use the ideas, rhetoric, and tools of ecosystem services science. *Ecosystem Services* 65: 101579.

[CR92] Tracy, S. J. 2019. *Qualitative Research Methods: Collecting Evidence, Crafting Analysis, Communicating Impact*. John Wiley & Sons, Incorporated, Newark, UNITED STATES.

[CR94] USDA Forest Service. 2004. The Large Tree Argument: The Case for Large-Stature Trees vs. Small-Stature Trees. Center for Urban Forest Research (Davis, CA) and Southern Center for Urban Forestry Research & Information (Athens, GA).

[CR95] Vogt, J. 2018. “Ships that pass in the night”: Does scholarship on the social benefits of urban greening have a disciplinary crosstalk problem? *Urban Forestry & Urban Greening* 32: 195–199.

[CR96] Wu, J., Y. Wang, S. Qiu, and J. Peng. 2019. Using the modified i-Tree Eco model to quantify air pollution removal by urban vegetation. *Science of the Total Environment* 688: 673–683. 31254833 10.1016/j.scitotenv.2019.05.437

[CR97] Xie, Y., S. Hirabayashi, S. Hashimoto, S. Shibata, and J. Kang. 2023. Exploring the Spatial Pattern of Urban Forest Ecosystem Services based on i-Tree Eco and Spatial Interpolation: A Case Study of Kyoto City. *Japan. Environmental Management* 72: 991–1005. 37382645 10.1007/s00267-023-01847-4

[CR98] Yao, N., C. C. Konijnendijk van den Bosch, J. Yang, T. Devisscher, Z. Wirtz, L. Jia, J. Duan, and L. Ma. 2019. Beijing’s 50 million new urban trees: Strategic governance for large-scale urban afforestation. *Urban Forestry & Urban Greening* 44: 126392.

[CR99] Young, R. F. 2013. Mainstreaming urban ecosystem services: A national survey of municipal foresters. *Urban Ecosystems* 16.

[CR100] Zoom. 2024. Zoom Video Communications Inc., San Jose, CA, USA.

