# Supplementary material for: The best laid plans: How do adopted city sustainability goals influence site-level action in urban forestry?
Source: Ambio. 2025 Sep 26;55(3):591–607. doi: 10.1007/s13280-025-02247-0 (PMC12868508; doi:10.1007/s13280-025-02247-0)
Supplement: Supplementary file 1 — Supplementary file1 (PDF 250 KB) [file 13280_2025_2247_MOESM1_ESM.pdf]

Ambio

Supplementary Information

This supplementary information has not been peer reviewed.

Title: The best laid plans: How do adopted city sustainability goals influences site-level action in urban forestry?

Table S1. Examples of ecosystem service goals within adopted municipal plans with the potential for application of urban forests as a strategy to address such goals. The presence of at least one goal, such as the ones below, in a publicly accessible plan document made a municipality eligible for inclusion in this study.

**Ecosystem service**	**Example of ecosystem service goal**
Climate change mitigation	"Reduce GHG emissions 80% below 1990 levels by 2050, with a long-term goal of becoming a net zero community" (p. 6).<br><br>City of Mississauga. (2021). *Climate Change Action Plan Progress Report.* https://www.mississauga.ca/wp-content/uploads/2020/09/30164812/Mississauga-Climate-Change-Action-Plan-Progress-Report-2021.pdf
Stormwater	"Goal 4: Leverage stormwater investments to help manage future flood risk from extreme rain and sea level rise. Future investments can alleviate flooding throughout the city" (p. 13).<br><br>City of New York. (2021). *New York City Stormwater Resiliency Plan.* https://www.nyc.gov/assets/orr/pdf/publications/stormwater-resiliency-plan.pdf
Urban heat	"N-1.4: Implement heat island mitigation strategies though development. Evaluate development codes for opportunities to encourage or require urban heat island mitigation strategies as part of new development or redevelopment." (p. 57).<br><br>Kansas City. (2022). *Climate Protection and Resiliency Plan.* https://www.kcmo.gov/home/showpublisheddocument/9912/638112897582470000
Biodiversity	"Explore incentives for and encourage developers to integrate natural biodiversity features (e.g., trees, wetlands) into development; Incorporate a Biodiversity Checklist (Appendix G) that will require developers to achieve a specified biodiversity target, but permit flexibility in how this can be achieved." (p. 85).<br><br>City of Surrey. (2014). *Biodiversity Conservation Strategy.* https://www.surrey.ca/sites/default/files/media/documents/Surrey_BCS_Report.pdf
Human health	"Maximize the role of parks and recreation in promoting healthy communities and lifestyles." (p. 164).<br><br>City of Austin. (2018). *Imagine Austin Comprehensive Plan*. https://www.austintexas.gov/sites/default/files/files/Imagine_Austin/IACP_2018.pdf

Table S2. Guide for semi-structured interviews with urban foresters at municipalities in the US & Canada, organized by section (Tracy 2019). Questions were often slightly adjusted, or different follow-up questions may have been asked during the interview, in response to participant and conversation.

**Section**	**Questions**
Opening	1.1 To begin, can you tell me briefly about your job history in urban forestry and how long have you worked in this field (urban forestry or arboriculture)?<br><br>1.2 How long have you been a [position title] at [City of____]?<br><br>1.3 What are your primary responsibilities and tasks in your current role?<br><br>1.4 To get an idea of the size of your program…<br><br>1.4a Do you have specific trees or a specific geographic area that you have authority over?<br><br>1.4b How many trees?<br><br>1.4c How large an area?<br><br>1.4d How many people are employed in urban forestry at your city? How many report to you?<br><br>1.5 What is your position's authority over decision making for these trees?<br><br>1.5a Does the city have full responsibility over all street tree management, or is that shared with anyone, such as adjacent property owners?
Generative	2.1 Specifically thinking about physical, or maintenance actions, what actions do you or your staff take in this phase? For example, we're interested in hearing about things like species selection, but not communication with a resident:<br><br>2.1a Pre-planting phase, such as species selection, siting where trees will be planted<br><br>2.1b Planting phase<br><br>2.1c Maintenance phase<br><br>2.1d What are the differences between management decisions made about young vs mature trees?<br><br>2.1e End of life, old trees, and removal phase

	2.2 When a decision needs to be made about street trees, what management objective or objectives is most important in deciding what to do?<br><br>2.3 Do you have any questions about this definition [Participant prompted with a definition of ecosystem services]?<br><br>2.4 Do you think it is part of your position's job duties to manage trees for any specific ecosystem services?<br><br>2.5 Given this definition, are you aware if your city has any goals related to ecosystem services like these, of any type? If so, what are they?<br><br>*(if yes, aware of some goals)*<br><br>2.5a What kind of documents or plans is that goal/are each of those included in? Urban forest planning documents, like a management plan or strategy? Climate action plan, municipal plan, resilience strategy?<br><br>2.5b Do you know how these goals got incorporated into these documents? Can you tell me about it?<br><br>*(If haven't heard of the plan they mentioned)*<br><br>2.5c Can I follow up to ask for a copy of the management plan or strategy?<br><br>2.5d What kind of input did you have in these plans?<br><br>*(if no, not aware of any ecosystem service goals)*<br><br>2.5e I have done some web searches and found some goals and plans I'd like to ask you about *(refer to list)*<br><br>2.5d Have you heard of this goal or plan before?<br><br>*(if the goal or plan explicitly mentions trees)*: Do you know how these goals got incorporated into these documents? Can you tell me about it?
Directive	3.1 To recap, we talked about [list the goals talked about] that your city has set. Overall, which of these goals is most important to manage for, in your position? Do you have the most responsibility to manage for?<br><br>3.1a Why is that?<br><br>3.1b Do you use any specific strategies to achieve that goal? What are they?

	3.2 How important is this goal compared to street tree management objectives you talked about before? *(prompt with the objective that was mentioned before, e.g., risk management)*<br><br>3.3 Now, using the example of the goal that was most relevant to your work, I want to talk really specifically about street tree management strategies. Part of my research is about bringing together all the possible ways that we adjust or change, in one way or another, the benefits of trees. So that when cities set goals, we can better align management to actually achieve those goals. Does that make sense?<br><br>3.4 Thinking back to the street tree management actions you talked through at the beginning, let's talk through this together:<br><br>3.4a Which decisions in the pre-planting phase could increase that benefit from street trees and help achieve that city goal?<br><br>3.4b Which decisions in the planting phase could increase that benefit and help achieve that city goal?<br><br>3.4c Which decisions in the young tree maintenance phase could increase that benefit and help achieve that city goal?<br><br>3.4d Which decisions in the mature tree maintenance phase could increase that benefit and help achieve that city goal?<br><br>3.4e Which decisions in the "end of life" phase could increase that benefit and help achieve that city goal?<br><br>3.5 Overall, do you think that street tree management in your city are aligned with the city's ecosystem service-related goals that we have discussed?<br><br>3.5a What do you do to align operations in urban forestry to the city-wide goals we discussed before?<br><br>3.5b Is there any particular data or tool that helps to do so? Does it assist in management decision making?<br><br>3.5c What are the obstacles and challenges to doing so?<br><br>3.5d Is there any information gaps you or your staff think you need to know how to manage trees for these city goals?<br><br>3.5e Do you feel like there is a goal that you don't know how to align to help achieve?
Closing	4.1 In an ideal world, what do you think are the best practices for cities to align street-level urban forest management actions to contribute to city-level ecosystem-service goals?

	4.1a What are barriers to implementing these best practices?<br><br>4.1b What would help cities be able to do this better?<br><br>4.2 Before we close, do you have any final thoughts to share about city-wide ecosystem service goals and street tree management?<br><br>4.3 That is the end of my questions! What did you think of this interview and my questions? Any feedback?

Table S3. Examples of the integration of urban forestry and ecosystem services in adopted non-urban forestry specific municipal plans. Urban forests are being included in municipal plans as a strategy for a broad array of ecosystem service goals, and with varying specificity.

**Ecosystem Service**	**Example**
Climate change mitigation	"Adopt energy efficiency and tree planting requirements to ensure rental housing is contributing to public health concerns associated with climate change" (p. 14).<br><br>City of Iowa City. (2020). *Acceleration Iowa City's Climate Actions.* https://www.iowa-city.org/WebLink/0/edoc/1944166/100%20Day%20Report%20-%20approved%20April%202020.pdf
Stormwater	"Use trees, vegetation, green stormwater infrastructure, amended soils, green roofs, and other low-impact development features to meet drainage needs and reduce the impacts of development." (p. 133).<br><br>City of Seattle. (2020). *Seattle 2035 Comprehensive Plan: Managing Growth to Become an Equitable and Sustainable City 2015-2035*. https://www.seattle.gov/Documents/Departments/OPCD/OngoingInitiatives/SeattlesComprehensivePlan/CouncilAdopted2020.pdf
Urban heat	"Goal 18: Establish a robust urban tree canopy and implement strategies to mitigate the UHI effect" (p. 36).<br><br>City of Louisville. (2013). *Sustain Louisville: Louisville Metro Sustainability Plan*. https://louisvilleky.gov/sustainability/document/sustainlouisvillepdf
Biodiversity	"Increase Biodiversity in Public Landscapes[...] Recommendation: Promote diverse and native plant species by requiring their use on city-owned property, including buildings, parks and sidewalks" (p. UE 1).<br><br>City of New York (n.d.). *NYC Green Codes Task Force: Full Proposals.* https://www.nyc.gov/html/gbee/downloads/pdf/urban_ecology.pdf
Human health	"P5.2 Develop more nature playgrounds and nature play features in parks. Create more opportunities to advance the physical and mental health benefits of children's play and exploration in nature." (p. 48).<br><br>City of Surrey. (2018). *Parks, Recreation & Culture Strategic Plan.* https://www.surrey.ca/sites/default/files/media/documents/PRCStrategicPlan.pdf
